# Supplementary material for: Linking neural population formatting to function
Source: bioRxiv. 2025 Jan 3:2025.01.03.631242. Preprint. [Version 1] doi: 10.1101/2025.01.03.631242 (PMC11722384; doi:10.1101/2025.01.03.631242)
Supplement: Supplement 2 [file NIHPP2025.01.03.631242v1-supplement-2.pdf]

bioRxiv preprint doi: https://doi.org/10.1101/2025.01.03.631242; this version posted January 4, 2025. The copyright holder for this preprint (which was not certified by peer review) is the author/funder, who has granted bioRxiv a license to display the preprint in perpetuity. It is made available under aCC-BY 4.0 International license.

Supplementary figures:

Supplementary Figure 1

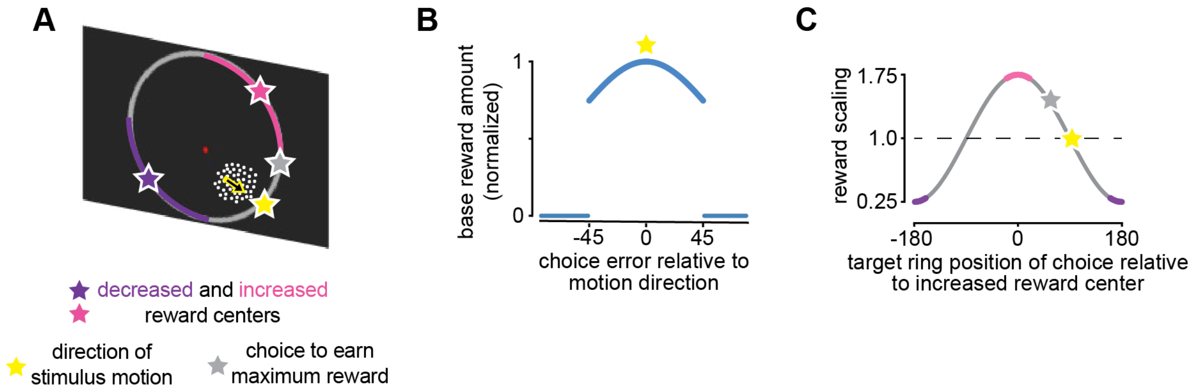

Supplementary Figure 1: The monkeys were rewarded based on the accuracy of their motion direction judgment, increased or decreased by a reward scaling factor associated with different choices. *A) Each trial required a motion direction judgment biased by reward scaling information indicated by the colored portions of the target ring. For the purposes of illustration, the reward scaling centers are noted with stars, the direction of stimulus motion is indicated with a yellow star and the choice associated with the largest reward is marked with a gray star. Both the motion direction and the reward condition were randomly interleaved from trial to trial, and the colors associated with increased or decreased reward varied in blocks with uncued changes. The raw reward amount varied across sessions, but rewards were always based on the (B) difference between the monkey's choice and the direction of visual motion (with a hard cut off at +- 45 degrees error) which was scaled by the values in (C). In this example reward condition, the increased reward center is the middle of the pink arc, and the decreased reward center is the middle of the purple arc.*

Supplementary Figure 2

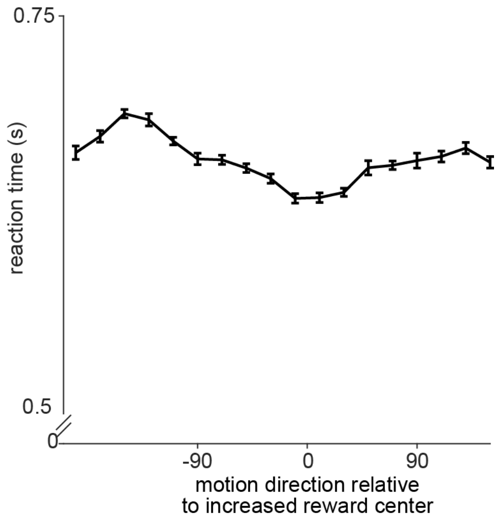

Supplementary Figure 2: Reaction times during the continuous motion estimation task were slightly faster when the motion direction was aligned with the increased reward center. *Mean reaction times (+- standard error of the mean) are plotted from 41,106 trials collected from 27 recording sessions (18 from monkey O). Reaction times are defined as the time from the onset of the visual stimulus to the time the monkey initiated the eye movement used to indicate a direction judgment. Reaction times were significantly modulated by the alignment between motion direction and the increased reward center (ANOVA, p<.01).*

bioRxiv preprint doi: https://doi.org/10.1101/2025.01.03.631242; this version posted January 4, 2025. The copyright holder for this preprint (which was not certified by peer review) is the author/funder, who has granted bioRxiv a license to display the preprint in perpetuity. It is made available under aCC-BY 4.0 International license.

Supplementary Figure 3

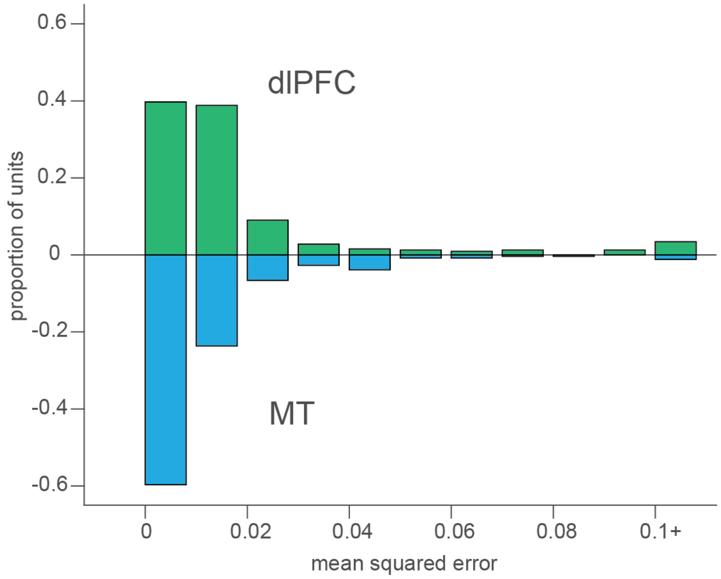

Supplementary Figure 3: dlPFC units exhibit more non-linear mixed selectivity to visual motion direction and reward information than MT. *The histograms of mean squared error of a motion direction tuning curve using a single scaling term to fit the two reward conditions (see Methods section 5.2) show greater error for dlPFC than MT (Wilcoxon rank sum test, $p<.01 \times 10^{-8}$). The histograms describe tuning curves for 258 MT units and 322 dlPFC units from 15 recording sessions in which there were simultaneous recordings in the two areas.*

bioRxiv preprint doi: https://doi.org/10.1101/2025.01.03.631242; this version posted January 4, 2025. The copyright holder for this preprint (which was not certified by peer review) is the author/funder, who has granted bioRxiv a license to display the preprint in perpetuity. It is made available under aCC-BY 4.0 International license.

Supplementary Figure 4

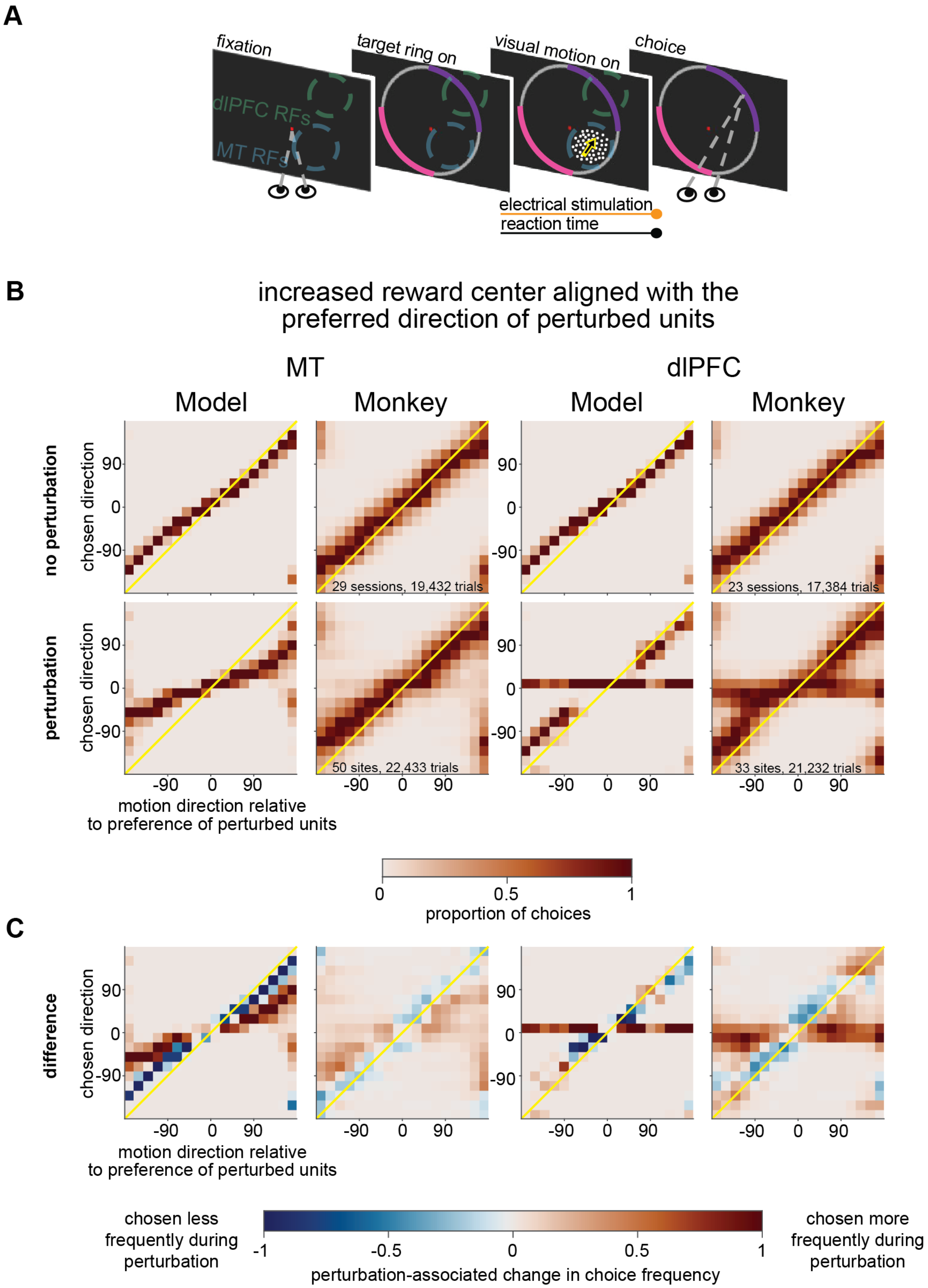

Supplementary Figure 4: Behavioral choices from model and monkey during perturbation experiments when the increased reward center was aligned with the preference of the perturbed units. A) *During monkey experiments, electrical stimulation coincided with the presentation of the visual stimulus.* ***B)*** *Summary of all choices by the model or monkeys when the increased reward center aligned with the*

bioRxiv preprint doi: https://doi.org/10.1101/2025.01.03.631242; this version posted January 4, 2025. The copyright holder for this preprint (which was not certified by peer review) is the author/funder, who has granted bioRxiv a license to display the preprint in perpetuity. It is made available under aCC-BY 4.0 International license.

preferred direction of the perturbed units. Data from 29 experimental sessions (comprising 50 sites; some sessions had stimulation on different electrodes on interleaved trials) of MT microstimulation (column 2), and 23 sessions (38 sites) of dlPFC microstimulation (column 4). Top row are choices during trials with no perturbation, bottom row are choices from trials with perturbation. All trial types, stimulus, and reward conditions were randomly interleaved within a session along with those shown in Supplementary Figure 5. Data are aligned to the tuning preference of the stimulated site, and sessions are only included in these analyses if the angular difference between that preference and the increased reward center was 30° or less. Data are normalized such that each column sums to 1. C) Plots reproduced from Figure 4B depicting the difference of the plots in (B).

Supplementary Figure 5

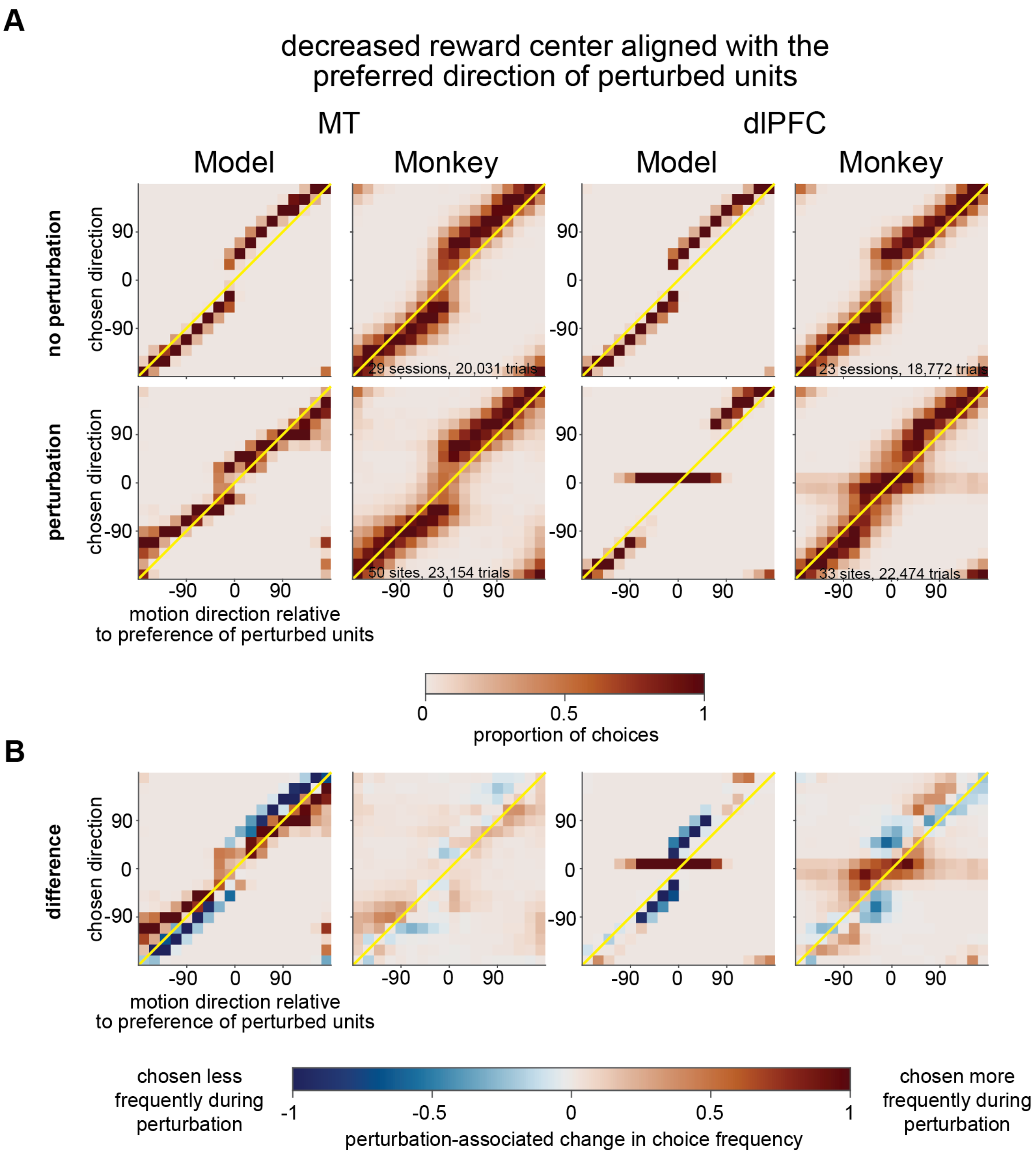

Supplementary Figure 5: Behavioral choices from model and monkey during perturbation experiments when the decreased reward center was aligned with the preference of the perturbed units. A) *Summary*

bioRxiv preprint doi: https://doi.org/10.1101/2025.01.03.631242; this version posted January 4, 2025. The copyright holder for this preprint (which was not certified by peer review) is the author/funder, who has granted bioRxiv a license to display the preprint in perpetuity. It is made available under aCC-BY 4.0 International license.

of all choices by the model or monkeys from the same experimental sessions and models as Supplementary Figure 4, on trials when the decreased reward center aligned with the preferred direction of the perturbed units. Conventions as in Supplementary Figure 4. B) Plots reproduced from Figure 4C depicting the difference of the plots in (A).

Supplementary Figure 6

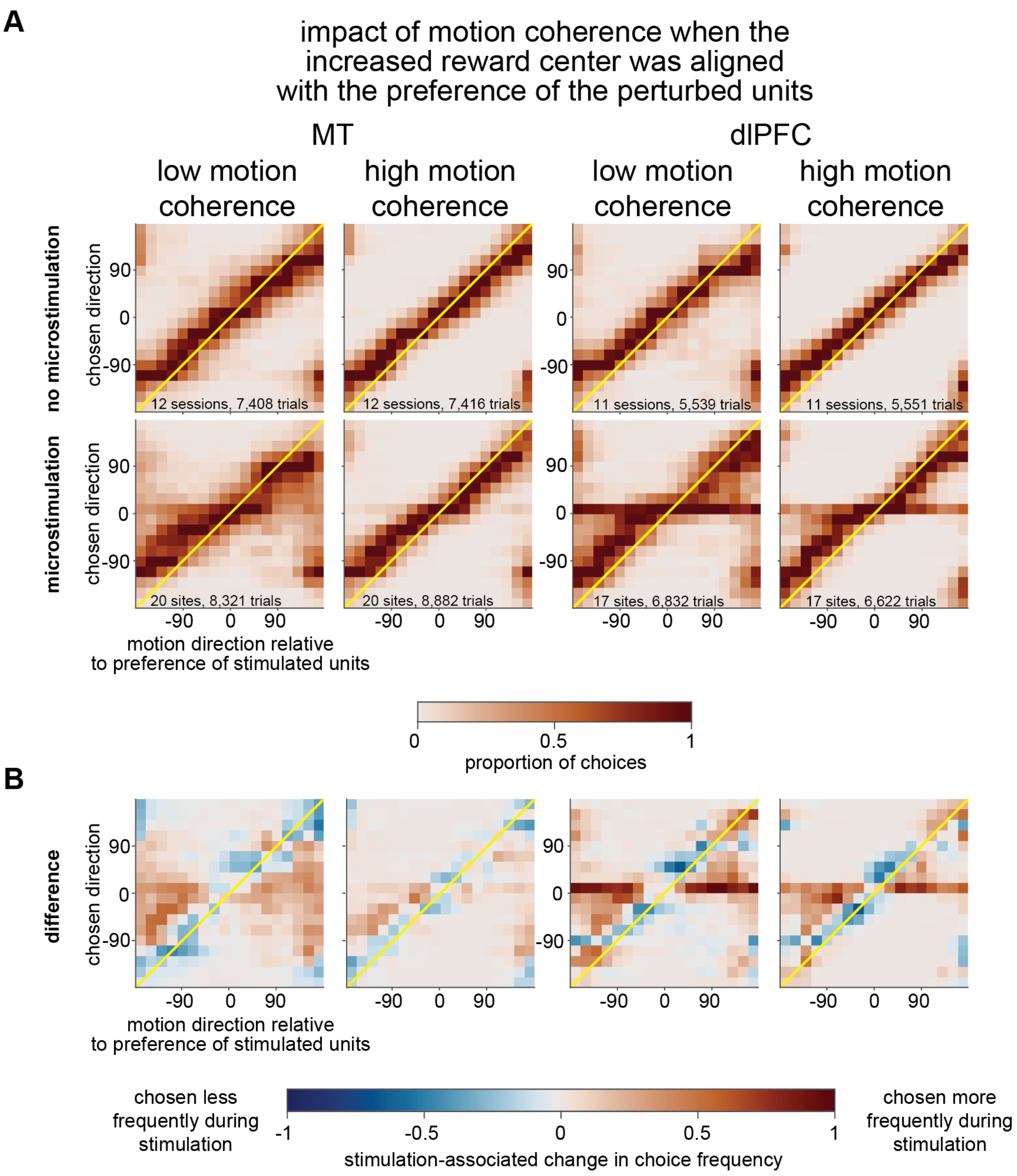

Supplementary Figure 6: The magnitude of, but not qualitative difference between, the behavioral impact of MT and dlPFC microstimulation depends on motion strength, on trials when the increased reward center was aligned with the preference of the perturbed units. *A) Summary of choices when the increased reward center aligned with the preferred directions of the microstimulated units during 12 experimental sessions (20 sites) of MT microstimulation and 11 sessions (17 sites) of dlPFC microstimulation using two motion coherences per session (low coherences were chosen between 15%*

bioRxiv preprint doi: https://doi.org/10.1101/2025.01.03.631242; this version posted January 4, 2025. The copyright holder for this preprint (which was not certified by peer review) is the author/funder, who has granted bioRxiv a license to display the preprint in perpetuity. It is made available under aCC-BY 4.0 International license.

and 20% coherence; high coherences were chosen between 35% and 50% coherence). The top row depicts trials with no microstimulation and the bottom row depicts trials with microstimulation. ***B)*** *Microstimulation-related difference in choices in both areas. The proportion of choices from A during non-microstimulation trials were subtracted from the proportion of choices during microstimulation trials at each coherence level. In MT, when the visual motion stimulus was at a lower coherence, microstimulation biased choices more strongly toward the preference of the stimulated site. In dlPFC, when the visual motion stimulus was lower coherence, microstimulation elicited more choices near the preference of the stimulated site.*

Supplementary Figure 7

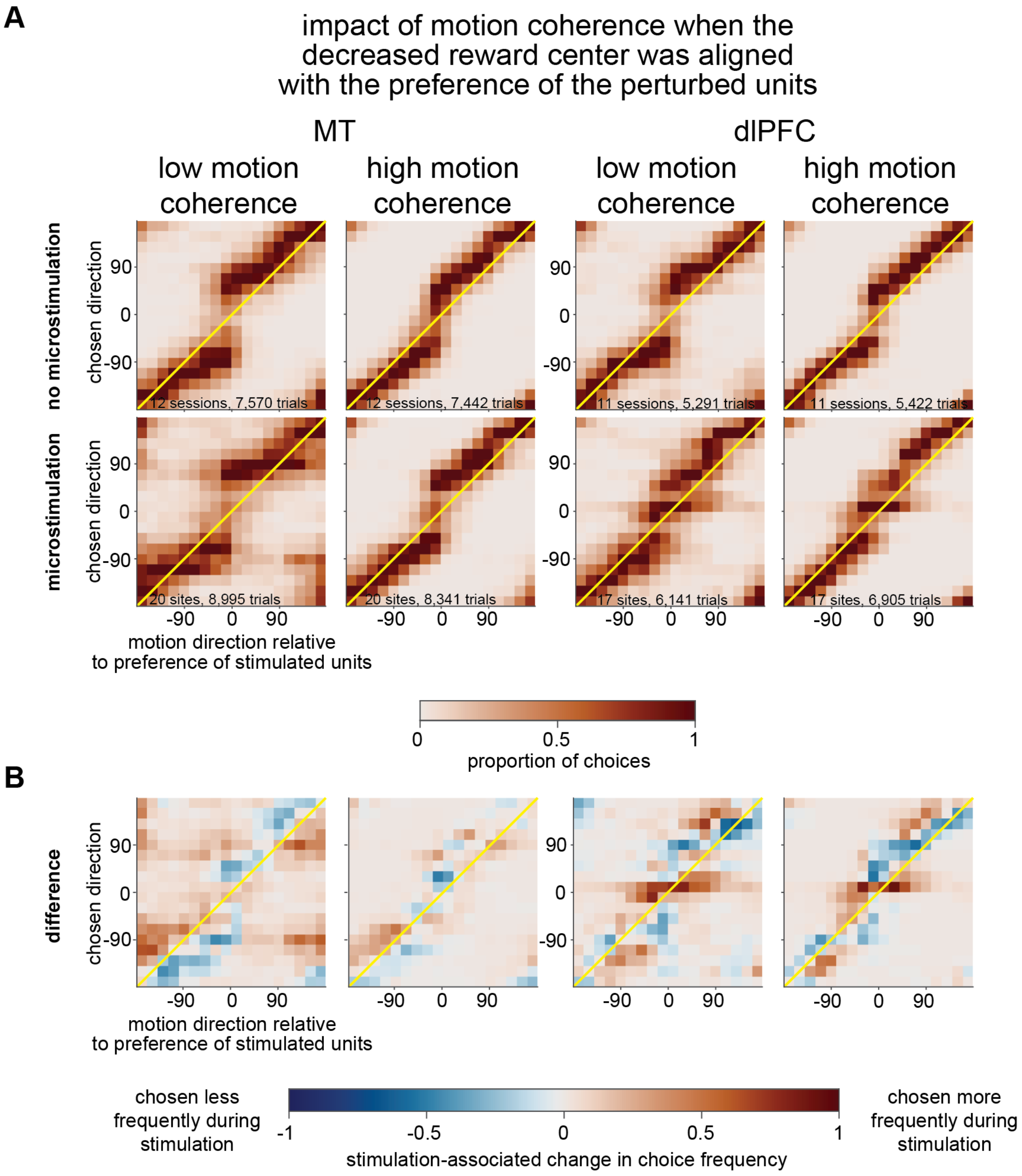

Supplementary Figure 7: The magnitude of, but not qualitative difference between, the behavioral impact of MT and dlPFC microstimulation depends on motion strength, on trials when the increased

bioRxiv preprint doi: https://doi.org/10.1101/2025.01.03.631242; this version posted January 4, 2025. The copyright holder for this preprint (which was not certified by peer review) is the author/funder, who has granted bioRxiv a license to display the preprint in perpetuity. It is made available under aCC-BY 4.0 International license.

reward center was aligned with the preference of the perturbed units. A) *Summary of choices on trials from the same recording sessions and models as Supplementary Figure 6 when the decreased reward center aligned with the preferred directions of the microstimulated units. Conventions as in Supplementary Figure 6A.* ***B)*** *Microstimulation-related difference in choices in both areas. Conventions as in Supplementary Figure 6B.*

bioRxiv preprint doi: https://doi.org/10.1101/2025.01.03.631242; this version posted January 4, 2025. The copyright holder for this preprint (which was not certified by peer review) is the author/funder, who has granted bioRxiv a license to display the preprint in perpetuity. It is made available under aCC-BY 4.0 International license.

Supplementary Figure 8

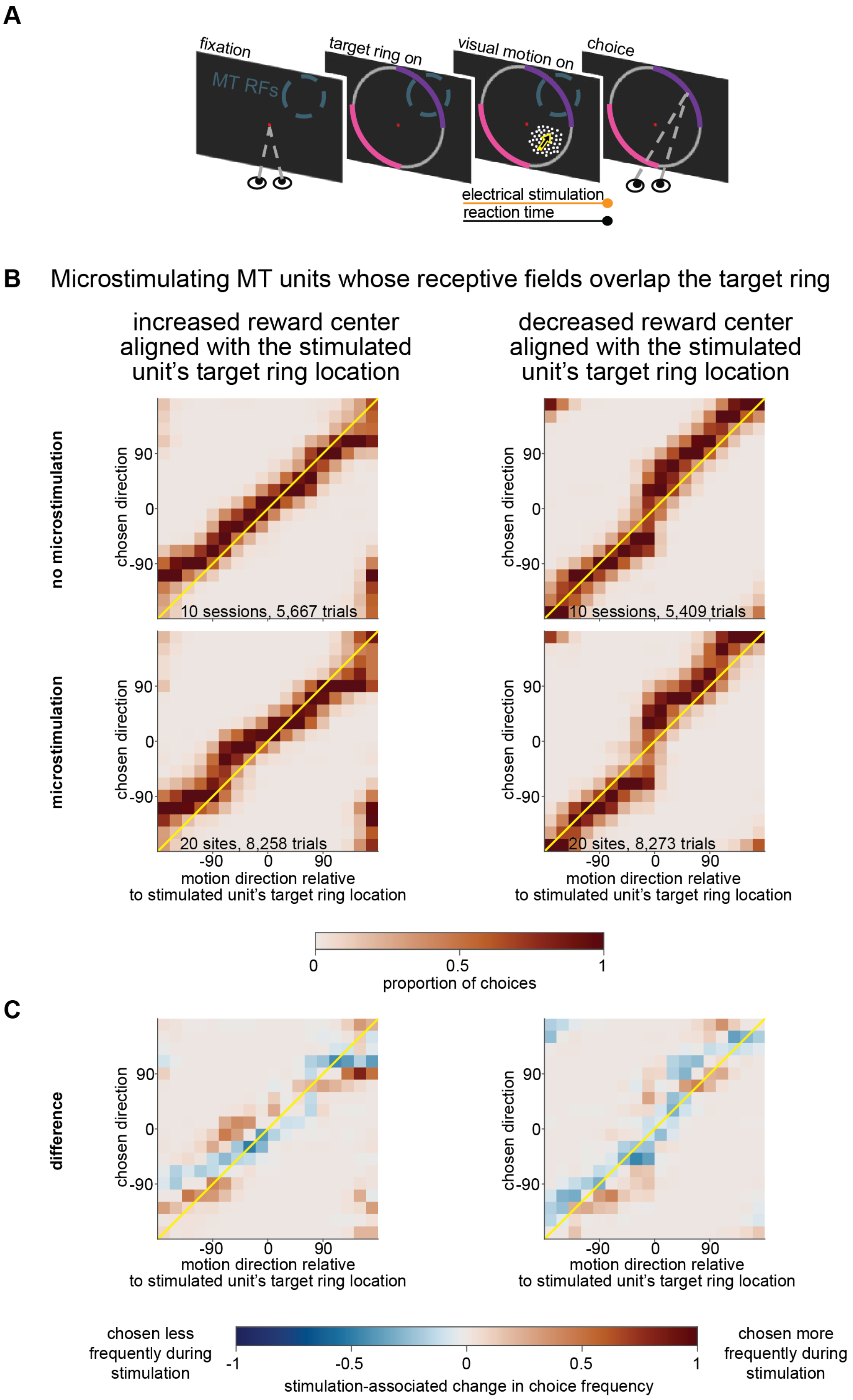

bioRxiv preprint doi: https://doi.org/10.1101/2025.01.03.631242; this version posted January 4, 2025. The copyright holder for this preprint (which was not certified by peer review) is the author/funder, who has granted bioRxiv a license to display the preprint in perpetuity. It is made available under aCC-BY 4.0 International license.

Supplementary Figure 8: Microstimulation in MT does not bias choices when the target ring, not the visual motion stimulus, overlaps the receptive fields of the microstimulated units. A) *As in all stimulation experiments, electrical stimulation coincided with the presentation of the visual stimulus, but unlike in most experiments (e.g. Figure 4), the receptive fields of the stimulated MT units overlapped the target ring, not the visual motion stimulus.* ***B)*** *Summary of choices from 10 experimental sessions (20 sites) of MT microstimulation when the receptive fields of the stimulated units overlapped the increased (left) or decreased (right) reward centers. Top row depicts trials with no microstimulation, bottom row depicts trials with microstimulation.* ***C)*** *Microstimulation-related difference in choices. The proportion of choices from B during non-microstimulation trials were subtracted from the proportion of choices during microstimulation trials for each reward condition. Choices were not biased by microstimulation in a way that is qualitatively similar to microstimulation in dlPFC (compare Figure 4B and C), even though in both cases, the receptive fields of the stimulated neurons overlapped part of the target ring.*
